# QTL Mapping of Low-Temperature Germination Ability in the Maize IBM Syn4 RIL Population

**DOI:** 10.1371/journal.pone.0152795

**Published:** 2016-03-31

**Authors:** Shuaidong Hu, Thomas Lübberstedt, Guangwu Zhao, Michael Lee

**Affiliations:** 1 The Key Laboratory for Quality Improvement of Agricultural Products of Zhejiang Province, College of Agriculture and Food Science, Zhejiang Agriculture and Forestry University, Lin’an, 311300, Zhejiang, China; 2 Department of Agronomy, Iowa State University, Ames, IA, 50011, United States of America; USDA Agricultural Research Service, UNITED STATES

## Abstract

Low temperature is the primary factor to affect maize sowing in early spring. It is, therefore, vital for maize breeding programs to improve tolerance to low temperatures at seed germination stage. However, little is known about maize QTL involved in low-temperature germination ability. 243 lines of the intermated B73×Mo17 (IBM) Syn4 recombinant inbred line (RIL) population was used for QTL analysis of low-temperature germination ability. There were significant differences in germination-related traits under both conditions of low temperature (12°C/16h, 18°C/8h) and optimum temperature (28°C/24h) between the parental lines. Only three QTL were identified for controlling optimum-temperature germination rate. Six QTL controlling low-temperature germination rate were detected on chromosome 4, 5, 6, 7 and 9, and contribution rate of single QTL explained between 3.39%~11.29%. In addition, six QTL controlling low-temperature primary root length were detected in chromosome 4, 5, 6, and 9, and the contribution rate of single QTL explained between 3.96%~8.41%. Four pairs of QTL were located at the same chromosome position and together controlled germination rate and primary root length under low temperature condition. The nearest markers apart from the corresponding QTL (only 0.01 cM) were umc1303 (265.1 cM) on chromosome 4, umc1 (246.4 cM) on chromosome 5, umc62 (459.1 cM) on chromosome 6, bnl14.28a (477.4 cM) on chromosome 9, respectively. A total of 3155 candidate genes were extracted from nine separate intervals based on the Maize Genetics and Genomics Database (http://www.maizegdb.org). Five candidate genes were selected for analysis as candidates putatively affecting seed germination and seedling growth at low temperature. The results provided a basis for further fine mapping, molecular marker assisted breeding and functional study of cold-tolerance at the stage of seed germination in maize.

## Introduction

Maize is one of the most widely grown crops in the world and ranks first for planting area in China. It is considered a cold-sensitive species with a relatively high temperature threshold for germination and vegetative growth [1]. Chilling damage is a common disaster at sowing season in many countries and regions and it has become one of the main environmental restrictions in maize production. In recent years, the frequent low temperature in early spring has resulted in the delay of sowing and the failure of normal emergence of maize seeds. Low seedling establishment ultimately leads to yield and seed quality decrease. It is, therefore, vital for maize breeding programs to improve tolerance to low temperatures at seed germination stage, called low-temperature germination ability, especially in temperate and at high altitudes in tropical and sub-tropical maize growing areas.

Quantitative trait locus (QTL) mapping of low-temperature germination ability has been conducted in rice, wheat, and soybean [2–9]. In rice, dozens of QTL of low-temperature germination ability were identified on 10 chromosomes using different mapping populations and it has been demonstrated that two QTL are useful for enhancing low-temperature germination ability in breeding programs. In maize, some traits at the seedling stage were conducted for mapping of QTL associated with chilling tolerance, such as chlorophyll fluorescence parameters, leaf greenness, leaf area, shoot dry weight, and shoot nitrogen content [10]. At the germination stage, germination rate and primary root length are the most important two traits in response to cold stress. However, little attention has been paid to their genetic analysis including QTL mapping. Therefore, it is imperative to carry out gene mapping of low-temperature germination ability in maize, which is the first step towards incorporating these traits in breeding programs.

The improvement of genetic resolution can be achieved by randomly intermating plants in the F2 generation prior to the derivation of mapping progeny [11]. In our study, the maize intermated B73×Mo17 (IBM) population was used. B73 and Mo17 represent two major heterotic groups in the U.S.: the Iowa stiff stalk synthetic population (BSSS) and Lancaster, respectively. The IBM mapping population was gradually constructed at Iowa State University since the 1990s [11]. Based on a B73×Mo17 F_2_ population, the IBM recombinant inbred line population (IBM Syn4 RIL, IBM Syn4 as short form) was generated after four generations of intermating and eight generations of inbreeding [11]. Recombination in IBM Syn4 is about 3.5 times increased compared to F_2_ -derived RILs. Therefore, IBM Syn4 is quite suitable for high density genetic linkage map construction, comparative genomics, QTL analysis and heterosis analysis [11].

In this study, a panel of 243 recombinant inbred lines of IBM Syn4 was used as QTL mapping population. The objectives of this study were: (i) to validate the low-temperature germination rate and primary root length of 243 Syn4 lines and their parents B73 and Mo17; (ii) to identify quantitative trait loci (QTL) controlling low-temperature germination rate and root length in the maize IBM Syn4; and (iii) to explore QTL which are colocalized in the same regions between germination rate and primary root length under the low temperature condition. This study will provide a basis for further fine mapping, molecular marker assisted breeding and functional study in low-temperature germination ability of maize.

## Materials and Methods

### Plant materials

Seed of 243 IBM Syn4 lines and their parents B73 and Mo17 were produced in summer 2006 by manual self-pollination of each plant at Agronomy Farm of Iowa State University (ISU), Ames, Iowa, USA located at 42°02′05″N, 93°37′12″W. This region has a humid continental climate and the average annual precipitation is 865.4 mm. After ripeness, seed was harvested and dried for seven days at ambient temperature and forced air at the Agronomy Research Center, ISU. Seed was subsequently stored at 4°C before use in the agronomy seed storage facility in the basement of Agronomy Hall, ISU. The initial average germination percentage of B73, Mo17 and IBM Syn4 population was 98%, 60%, and 82% at 28°C, respectively.

### Germination experiments

Germination experiments were performed in a growth chamber at Iowa State University in 2014 at 12°C /16h–18°C /8h daily cycles in the dark at 60% relative humidity for 7 d. According to practical maize production in China, sowing begins when the soil temperature reaches 15°C in early spring. The soil temperature may be 12°C at night, while it may be 18°C during the daytime. Therefore, to simulate the field conditions, alternating low temperatures were adopted in our experiment. In order to amplify the effect of low temperature, 12°C was used for double time of 18°C done every day. A control germination experiment was performed under optimum temperature condition (28°C/24h) for calculation of germination rates. Kernels were sown in moist brown germination paper (Size: 30 cm × 30 cm, Anchor Ltd., USA) and another sheet of humid paper was used as cover. Then, the germination paper was rolled and put erectly in a sealed plastic bag. After 7 d incubation, primary root length of each germinated seed under low-temperature condition was measured by a straight ruler and germination rates under both conditions of low temperature and optimum temperature were calculated according to the International Rules for Seed Testing [12]. The germination test was conducted in three replicates for each line. Fifteen seeds per line were used for each replicate.

Significant differences of germination rate and primary root length under low temperature condition between the two parental lines B73 and Mo17 were detected using Student's *t* test (*p* = 0.01). Data of 243 IBM lines were analyzed using analysis of variance (ANOVA) implemented in the PROC GLM program in SPSS 12.0 (SPSS, Inc., Chicago). Heritabilities (*H*^*2*^, %) for each trait were calculated, fitting the effects of genotype (*G*) and environment (*E*), as *H*^*2*^ = *σ*^*2*^_*G*_/ (*σ*^*2*^_*G*_ +*σ*^*2*^_*E*_/*r*) × 100, where *H*^*2*^ is broad sense heritability, *σ*^*2*^_*G*_ is genotypic variance, *σ*^*2*^_*E*_ is error variance, and *r* is the number of replicates [13]. The coefficients of variation (CV, %) for each trait were calculated as follows: $\text{CV}=\text{s }/\;\bar{\text{x}}$, where s is the standard deviation.

### DNA marker analysis and genetic linkage map construction

DNA marker data used for genetic linkage map construction were retrieved from the MaizeGDB website (http://www.maizegdb.org/ancillary/qtl/ibm302cross.np). In this study, 1339 DNA markers were used. Marker information marked “A” correspond to the genotype of B73, “B” to that of Mo17, and “-” represents a missing value. The marker order was determined using MAPMAKER/EXP3.0 and the genetic map was constructed with the software newly developed by Liu et al. [14]. Marker loci were partitioned into linkage groups (LGs) based on their location in the maize genome. Next, the modified logarithm of odds (MLOD) scores between DNA markers were calculated to further confirm the robustness of markers for each LG. Markers with MLOD scores <5 were filtered prior to ordering. To ensure efficient construction of the high-density and high-quality map, the HighMap strategy as described by Liu et al. [14] was utilized to order the DNA markers and correct genotyping errors within LGs.

### QTL analysis

QTL analysis was carried out using the above linkage map with 1339 markers. Two methods of composite interval mapping (CIM) and multiple interval mapping (MIM) were employed to identify QTL and to estimate their effects throughout the whole genome [15, 16]. Because similar results were obtained, only those from CIM are shown. QTL Cartographer 1.17 was used, and model 6 of the Zmapqtl program module was adopted. The genome was scanned at 2 cM intervals and the window size was set at 10 cM. The logarithm of odds (LOD) threshold values for each trait were determined by 1,000 permutations at a *p* = 0.05 level [17]. QTL positions were assigned underneath maximal LOD scores. The calculations were performed with QTL Cartographer 1.17 developed by Basten et al. [16]. Additive effects of the detected QTL were estimated using Zmapqtl. The *R*^*2*^ value (coefficient of determination) from this analysis indicates the percentage of phenotypic variance explained by marker genotypes at a QTL. The procedures of QTL analysis used in this study are described in more detail in S1 Table.

## Results

### Phenotypic analysis for germination-related traits under both conditions of low temperature and optimum temperature

Three traits related to germination ability were measured (Table 1): optimum-temperature germination rate (OTGR), low-temperature germination rate (LTGR) and low-temperature primary root length (LTPRL). The parental lines B73 and Mo17 were significantly different for OTGR (*p* = 0.05), LTGR (*p* = 0.01) and LTPRL (*p* = 0.01). There were significant correlations between OTGR and LTGR (r = 0.681, *p* = 0.01), LTPRL (r = 0.679, *p* = 0.01). Furthermore, LTGR and LTPRL showed a closer significant correlation (r = 0.896, *p* = 0.01), suggesting that germination-related traits under low temperature condition were more likely to be controlled by the same genetic factors.

**Table 1:** Germination related traits of B73, Mo17 and Syn4 population and their heritabilities under low and optimal temperature conditions.

Traits^a^	Parent^b^		Syn4 population			
	B73	Mo17	Range	Mean	CV (%)^c^	*H*^*2*^ (%)^d^
OTGR (%)	97.8±3.8A	60.0±6.7B	4.4–100.0	81.7	25.9	88.5
LTGR (%)	95.6±3.8A	35.6±3.8B	0.0–100.0	52.6	58.1	88.3
LTPRL (mm)	26.7±1.0A	8.1±2.1B	0.2–36.0	11.3	54.7	94.5

^a^OTGR, LTGR, LTPRL represents optimum-temperature germination rate, low-temperature germination rate, low-temperature primary root length, respectively.

^b^Means with the standard deviation of the parental lines that followed by the different upper-case letter significantly differ by Student's *t* test at 1% level of significance, respectively.

^c^CV: coefficient of variation.

^d^*H*^*2*^: heritability.

**Fig 1:**
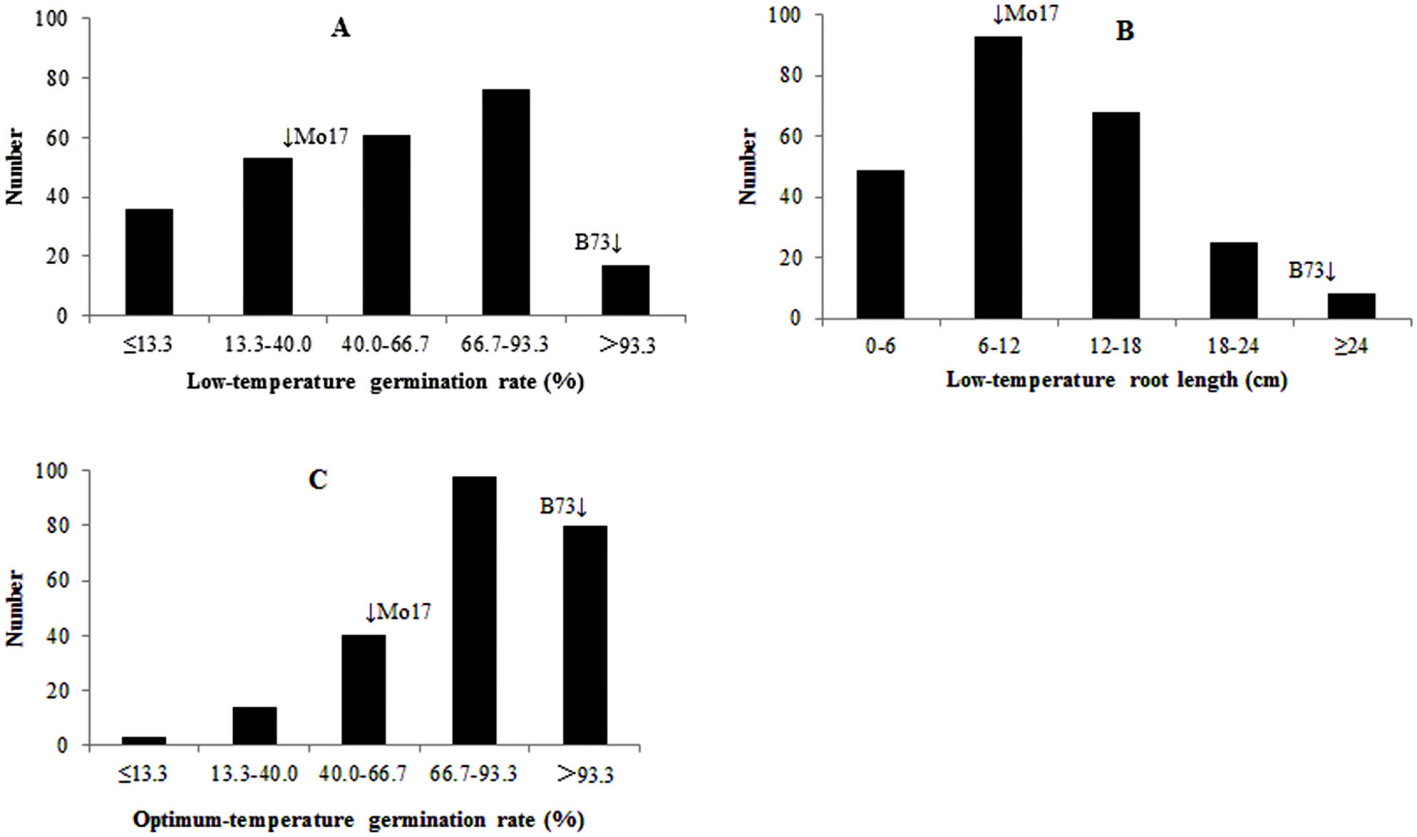
Histograms of frequency distributions of germination-related traits under low and optimum temperature in the IBM Syn4 population. A. Low-temperature germination rate, B. Low-temperature primary root length, C. Optimum-temperature germination rate.

The average values of LTGR and LTPRL within IBM Syn4 were closer to those of Mo17 than B73 (Table 1). However, the contrary trend was found for OTGR. OTGR had a lower coefficient of variation (CV, %) value than LTGR and LTPRL. CVs of OTGR, LTGR, and LTPRL were 25.9%, 58.1%, and 54.7%, respectively. Furthermore, LTPRL had a higher heritability (*H*^*2*^) than OTGR and LTGR. *H*^*2*^ estimates of LTPRL, OTGR, and LTGR were 94.5%, 88.5% and 88.3%, respectively. The results suggested that LTPRL was less affected by environmental factors than LTGR. Germination rate under both low and optimum temperature followed negatively skewed distributions, while primary root length under low temperature condition followed a positively skewed distribution (Fig 1). Peak germination rates at both temperatures were higher than the mean of the IBM Syn4 population, whereas primary root length at low temperature was reduced. Furthermore, only a single peak appeared in the frequency distribution histograms for each trait, suggesting that the three germination-related traits might be controlled by several QTL with a small genetic effects.

Under low temperature condition, within 243 lines of IBM Syn4, primary root lengths after seven days of five lines exceeded 25.0 cm, whereas those of three lines were below 1.0 cm. Germination rates of 27 lines exceeded 98%, whereas those of 16 lines were below 2%. In general, seed of 10 lines had stronger germination ability (germination rate ≥ 95% and primary root length ≥ 20.0 cm) to tolerate low temperatures, whereas seeds of 12 lines had weaker germination ability to tolerate low temperatures (germination rate ≤ 10% and primary root length ≤ 3.0 cm).

### Construction of a molecular linkage map

In total, 1339 DNA markers were included in the construction of a molecular linkage map. There were 229, 128, 162, 131, 128, 109, 116, 107, 126, 103 DNA markers located on chromosomes 1, 2, 3, 4, 5, 6, 7, 8, 9, and 10, respectively. They were used to construct 10 linkage groups (S1–S10 Figs). The total length of the IBM Syn4 linkage map was 6242.7 cM with an average interval size of 4.63 cM.

### QTL analysis for germination-related traits under both conditions of low temperature and optimum temperature

**Fig 2:**
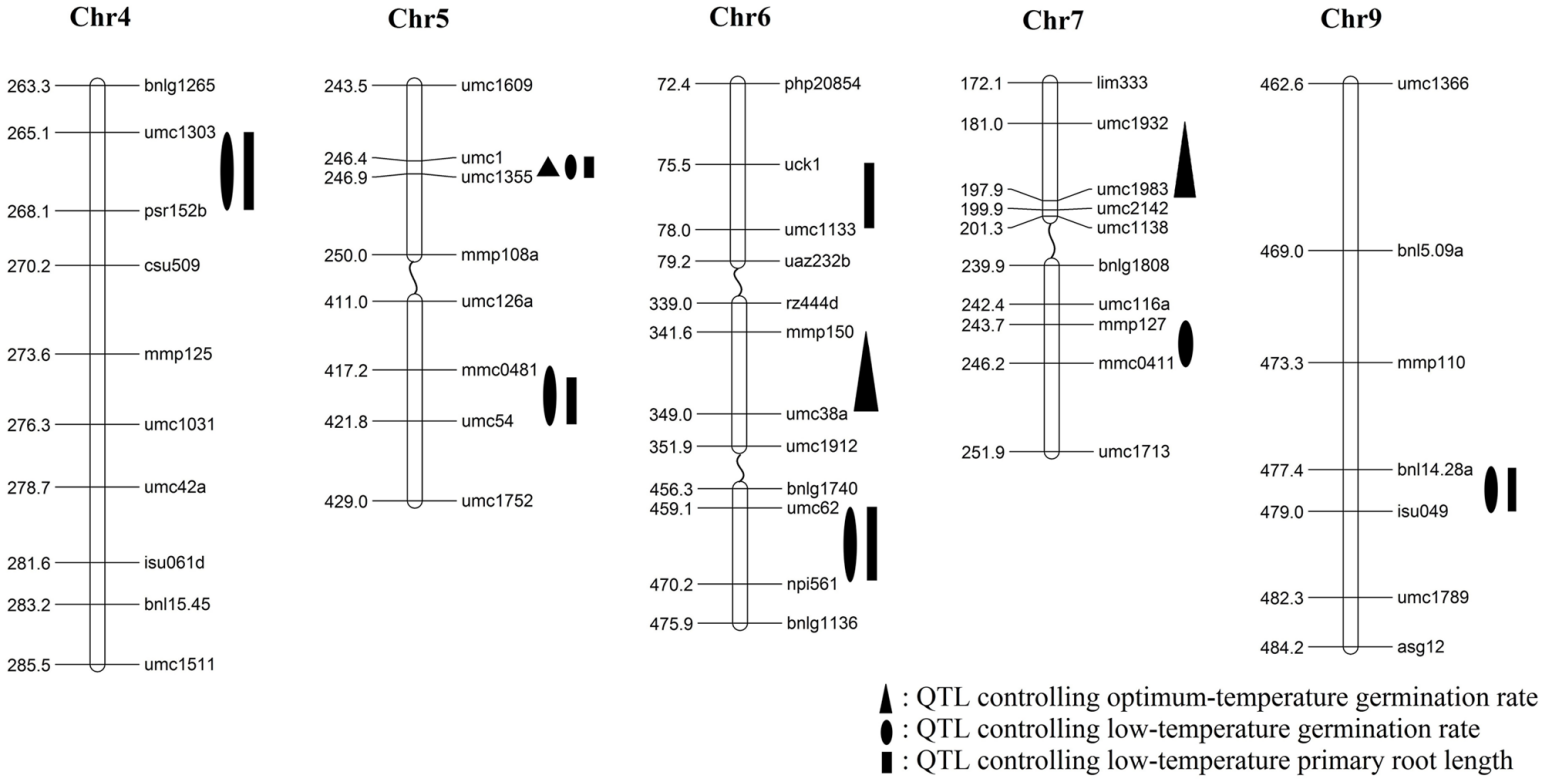
Chromosomal location of quantitative trait loci (QTL) for germination ability under low and optimum temperature conditions in IBM Syn4 population.

The genotypic data (S2 Table) were retrieved from the MaizeGDB website (http://www.maizegdb.org/ancillary/qtl/ibm302cross.np). QTL were detected based on LOD thresholds after permutation tests. The LOD thresholds for optimum-temperature germination rate (OTGR) varied from 3.72 to 4.72. Only three QTL controlling OTGR were detected on chromosomes 5, 6, 7 (Table 2; Fig 2). These QTL explained 5.75–7.78% of the phenotypic variation. The LOD thresholds for low-temperature germination rate (LTGR) varied from 3.12 to 9.66. A total of six QTL controlling LTGR were detected on five chromosomes (Table 2; Fig 2). Of the six QTL, two QTL were located on chromosome 5, the other four QTL was located on chromosomes 4, 6, 7, and 9. These QTL explained 3.39–11.29% of the phenotypic variation. *qLTGR5-1* had the highest LOD values and the highest contribution to phenotypic variance. The QTL region from B73 background had a positive effect on LTGR (Table 2). The LOD thresholds for low-temperature primary root length (LTPRL) varied from 3.33 to 6.66. A total of six QTL controlling LTPRL were detected on four chromosomes (Table 2; Fig 2). Of the six QTL, two QTL were located on chromosome 5, two QTL were located on chromosome 6, and the other two QTL were located on chromosomes 4 and 9. These QTL explained 3.96–8.41% of the phenotypic variation. *qLTPRL9-1* had the highest LOD values and explained most of the phenotypic variation. The QTL region from the B73 background had a negative effect on LTPRL (Table 2).

**Table 2:** QTL detected for controlling germination ability under low and optimum temperature in the IBM Syn4 population.

QTL^a^	Peak position (cM)	Bin^b^	Nearest marker	Left border (cM)	Right border (cM)	LOD Score^c^	Additive Effect^d^	R^2^ (%)^e^
*qOTGR5-1*	246.41	5.03	umc1	246.4	246.9	4.72	-5.56	6.37
*qOTGR6-1*	342.61	6.05–6.06	mmp150	341.6	349	3.88	5.18	5.75
*qOTGR7-1*	190.01	7.02	umc1983	181	197.9	3.72	6.33	7.78
*qLTGR4-1*	265.11	4.05	umc1303	265.1	268.1	3.12	0.88	3.39
*qLTGR5-1*	246.41	5.03	umc1	246.4	246.9	9.66	-1.59	11.29
*qLTGR5-2*	417.21	5.06	mmc0481	417.2	421.8	5.12	1.21	5.7
*qLTGR6-1*	459.11	6.07	umc62	459.1	470.2	3.42	0.95	3.89
*qLTGR7-1*	244.71	7.02	mmp127	243.7	246.2	4.77	1.15	5.72
*qLTGR9-1*	477.41	9.06	bnl14.28a	477.4	479	4.61	1.07	5.25
*qLTPRL4-1*	265.11	4.05	umc1303	265.1	268.1	3.33	1.26	3.96
*qLTPRL5-1*	246.41	5.03	umc1	246.4	246.9	4.42	-1.47	5.39
*qLTPRL5-2*	419.21	5.06	mmc0481	417.2	421.8	6.08	1.91	8.11
*qLTPRL6-1*	75.51	6.01	uck1	75.5	78	5.17	-2.48	7.03
*qLTPRL6-2*	459.11	6.07	umc62	459.1	470.2	4.1	1.47	5.17
*qLTPRL9-1*	477.41	9.06	bnl14.28a	477.4	479	6.66	1.83	8.41

^a^OTGR, LTGR, LTPRL represents optimum-temperature germination rate, low-temperature germination rate, low-temperature primary root length, respectively.

^b^Chromosome Bins of the marker and position taken from IBM 2008.

^c^LOD: Log10-likelihood value.

^d^Additive effect: The negative value of additive effect indicates the allele from B73 is positive.

^e^R^2^: coefficient of determination, which represents the percentage of phenotypic variance explained by a putative QTL.

Three QTL were located at the same chromosome position and together controlled three germination-related traits (Table 2, Fig 2). *qOTGR5-1*, *qLTGR5-1*, and *qLTPRL5-1* were detected at 246.41 cM on chromosome 5, which was near umc1 (246.4 cM). Alleles from B73 background promoted germination ability under both conditions of low temperature and optimum temperature (Table 2). In addition, four pairs of QTL were separately located at the same chromosome position and each pair controlled both germination rate and primary root length under low temperature condition (Table 2, Fig 2). *qLTGR4-1* and *qLTPRL4-1* were detected at 265.11 cM on chromosome 4 near umc1303 (265.1 cM). *qLTGR6-1* and *qLTPRL6-2* were detected at 459.11 cM on chromosome 6, near to umc62 (459.1 cM). *qLTGR9-1* and *qLTPRL9-1* were detected at 477.41 cM on chromosome 9, close to bnl14.28a (477.4 cM). *qLTGR5-2* and *qLTPRL5-2* were located near mmc0481 (417.2 cM) on chromosome 5.

## Discussion

Early sowing can have agronomic advantages such as earlier harvest and greater yield [18]. It is, therefore, an important breeding objective to improve maize cold tolerance at germination stage. There are genetic differences of low-temperature germination ability among different maize genotypes. Seedling cold tolerance has been studied at an agronomic and physiological level with respect to number of surviving plants, plant weight, leaf greenness, leaf area, chlorophyll fluorescence parameters, nitrogen content, and anthocyanin content [1, 10, 19]. However, little is known about maize QTL involved in low-temperature germination ability. So far, Hund et al. [20] reported QTL controlling root and shoot traits of maize seedlings under cold stress.

As is well known, the radicle firstly protrudes from the seed coat and subsequent growth is positively correlated with germination. Therefore, primary root length and germination rate play a vital role in low-temperature germination ability in maize. Under low temperature condition, primary root length and germination rate had higher heritabilities and relevancy in our study than in the study by Hund et al. [20]. Therein, four QTL were detected for primary root length and one QTL was detected for germination rate using a linkage map comprising 135 markers in Hund's study [20]. Compared with our results, there were two QTL of germination-related traits detected at the same chromosome. One QTL controlling LTGR was located at 110 cM on chromosome 4, 155.11 cM apart from *qLTGR4-1* and another QTL controlling LTPRL was located at 119 cM on chromosome 4, 146.11 cM apart from *qLTPRL4-1* in our study. Additional QTL controlling low-temperature germination ability were thus identified in our study.

The different results comparing the study of Hund et al. [20] and ours might be caused by different testing conditions, such as type and number of molecular markers, mapping populations, as well as temperature conditions. Firstly, there were 1339 DNA markers employed in our study, whereas there were only 135 DNA markers employed in the study of Hund et al. [20]. A denser linkage map contributed to more precise QTL localization in our study. Secondly, 243 recombinant inbred lines of the IBM Syn4 population were analyzed in our study, whereas only 168 F2:4 families of the cross Lo964 × Lo1016 were analyzed in the study of Hund et al. [20]. In our study, the IBM Syn4 RIL population was chosen because B73 and Mo17 represent the main heterotic groups of temperate elite maize in the U.S., and an extensive genetic map is available. Furthermore, the IBM population offers a high genetic resolution and power of QTL detection [19]. So, the differences in numbers of markers and individuals explained the accuracy and precision in the QTL identified. In addition, to simulate the field conditions, an alternating low temperature regime (12°C/16h, 18°C/8h) was adopted during seed germination in our study. In the study of Hund et al. [20], seeds were only imbibed for 12 h at 16°C before sowing and then kept at a high constant temperature (25°C) during seed germination. Finally, B73 and Mo17 significantly differed in germination rate under low temperature conditions [19]. When optimum temperature was applied, 3 QTL were identified in our study while no QTL were detected in the study of Rodríguez et al. [19].

The high resolution linkage map not only contributes to QTL mapping of low-temperature germination ability and understanding of its molecular basis, but will also to be helpful for breeding of cold-tolerant cultivars via marker-assisted selection (MAS). In rice, Hyun et al. [21] developed several SNP markers of low-temperature germination ability for evaluation of rice germplasm based on the major effect QTL *qLTG3-1*. In our study, there were four pairs of QTL located in the same chromosome position, which together controlled germination rate and primary root length of maize under low temperature condition. The genetic distances were very close (only 0.01 cM) between QTL and DNA markers. The corresponding four DNA markers closely linked with their QTL of low-temperature germination ability were umc1303 (265.1 cM) on chromosome 4, umc1 (246.4 cM) on chromosome 5, umc62 (459.1 cM) on chromosome 6, and bnl14.28a (477.4 cM) on chromosome 9. Therefore, these four DNA markers are useful tools using MAS to predict cold-tolerance in breeding programs.

In our study, a total of 15 QTL were detected in three germination-related traits. Because of common QTL, there were only nine distinct intervals controlling optimum-temperature germination rate, low-temperature germination rate or low-temperature primary root length. A total of 3155 candidate genes were extracted from the nine intervals based on B73_RefGen_v3 in Maize Genetics and Genomics Database (http://www.maizegdb.org) (S3 Table). Three QTL (*qOTGR5-1*, *qLTGR5-1*, *qLTPRL5-1*) near umc1 were identified at the same locus (60,994,667~64,729,128 bp) on chromosome 5. The *GRMZM2G325653* gene (64,674,608~64,681,233 bp) is expressed in seedling coleoptile, primary shoot system, juvenile vascular leaf, among others [22]. It encodes a mildew resistance locus (MLO)-like protein and its homolog in Arabidopsis was expressed during seedling growth [23]. Two QTL (*qLTGR4-1*, *qLTPRL4-1*) near umc1303 were located in the same region (42,146,791~65,900,096 bp) on chromosome 4. A candidate gene *GRMZM2G377165* (42,144,894~42,148,125 bp) is a putative serrate-related C2H2 zinc-finger family protein. According to the study of Prigge and Wagner [24], the homologous gene in Arabidopsis is required for normal shoot development such as taller meristems, alterations in phase transition, phyllotaxy and branching. Two QTL (*qLTGR5-2*, *qLTPRL5-2*) near mmc0481 were detected in the same position (192,286,059~192,786,734 bp) on chromosome 5. The gene *GRMZM2G398807* (192,613,610~192,614,489 bp) coding a cortical cell-delineating protein was expressed in primary root and perhaps participates in the physiological process of radicle protrusion [22, 23]. Two QTL (*qLTGR6-1*, *qLTPRL6-2*) near umc62 were detected in the same position (164,802,199~165,689,335 bp) on chromosome 6. A predicted gene *GRMZM2G154595* (165,631,098~165,635,858 bp) was expressed in some germination-related organs such as embryo, primary root, coleoptile, stem internode and translated to malate dehydrogenase. It is well known that some key enzymes (malate dehydrogenase, etc.) in respiratory metabolism and TCA cycle are not only a fundamental metabolic pathway for energy production, but also provide the substrates for cell synthesis and growth during seed germination [25]. Two QTL (*qLTPRL9-1*, *qLTGR9-1*) near bnl14.28a are positioned in the same region (147,818,369~147,658,717 bp) on chromosome 9. The product of a candidate gene *GRMZM2G154149* (147,664,617~147,669,617 bp) is a putative non-phototropic hypocotyl 3 (NPH3) family protein isoform and its homologs in Arabidopsis and rice are involved in auxin-mediated organogenesis such as hypocotyl [22, 23]. These predicted genes may affect seed germination and seedling growth especially at low temperature.

## Supporting Information

S1 Fig Linkage group in Chr1. (PNG)

S2 Fig Linkage group in Chr2. (PNG)

S3 Fig Linkage group in Chr3. (PNG)

S4 Fig Linkage group in Chr4. (PNG)

S5 Fig Linkage group in Chr5. (PNG)

S6 Fig Linkage group in Chr6. (PNG)

S7 Fig Linkage group in Chr7. (PNG)

S8 Fig Linkage group in Chr8. (PNG)

S9 Fig Linkage group in Chr9. (PNG)

S10 Fig Linkage group in Chr10. (PNG)

S1 Table The detailed procedures of QTL analysis. (DOCX)

S2 Table The 243 lines' genotypes for IBM Syn4 RIL population. (XLSX)

S3 Table A list of predicted genes in each confidence interval. (XLSX)
