# Supplementary figures and images for: QTL Mapping of Low-Temperature Germination Ability in the Maize IBM Syn4 RIL Population
Source: PLoS One. 2016 Mar 31;11(3):e0152795. doi: 10.1371/journal.pone.0152795 (PMC4816396; doi:10.1371/journal.pone.0152795)

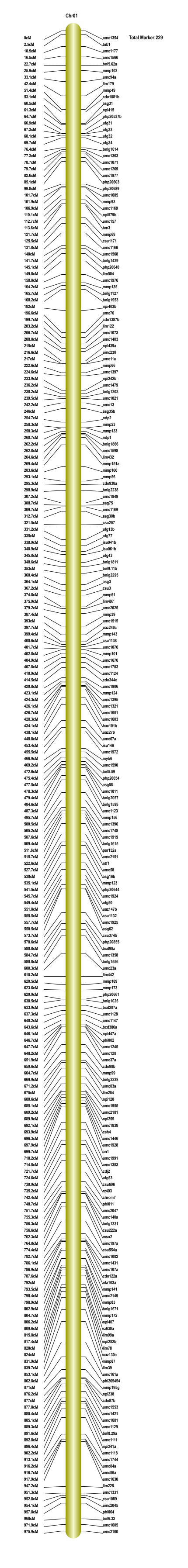

Supplement: S1 Fig — (PNG) [file pone.0152795.s001.png]

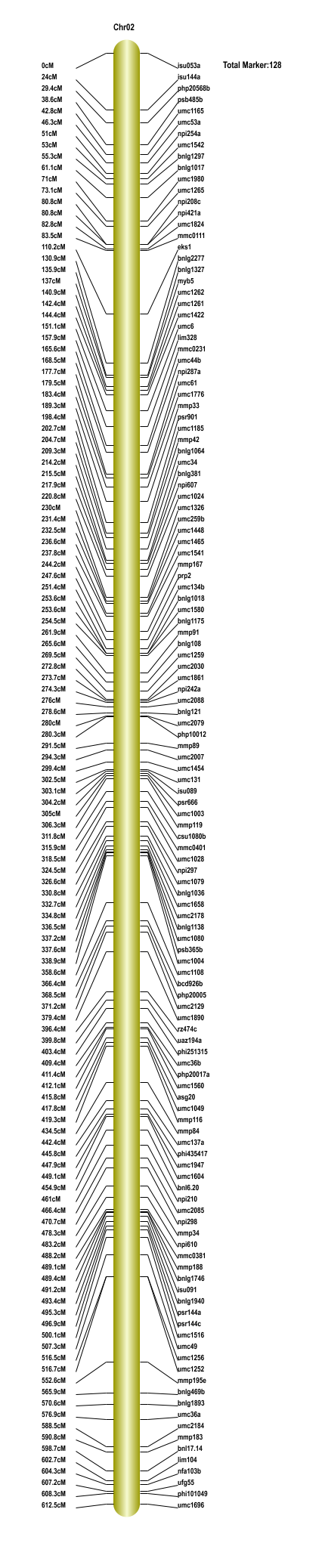

Supplement: S2 Fig — (PNG) [file pone.0152795.s002.png]

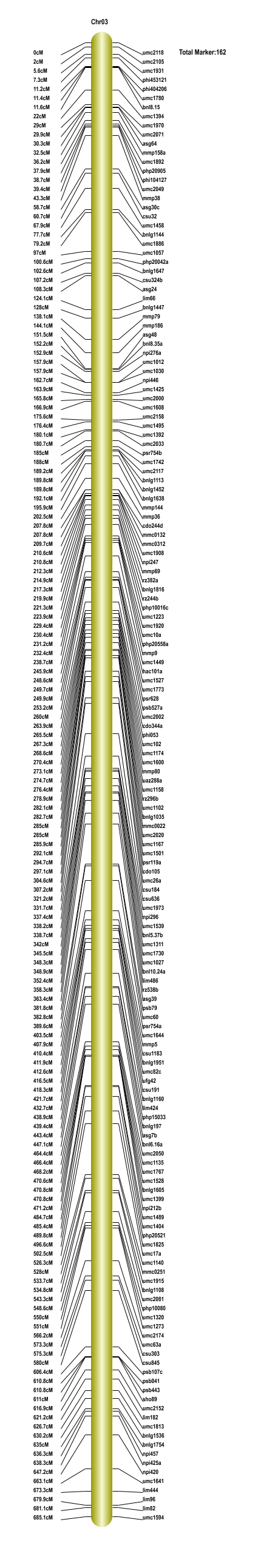

Supplement: S3 Fig — (PNG) [file pone.0152795.s003.png]

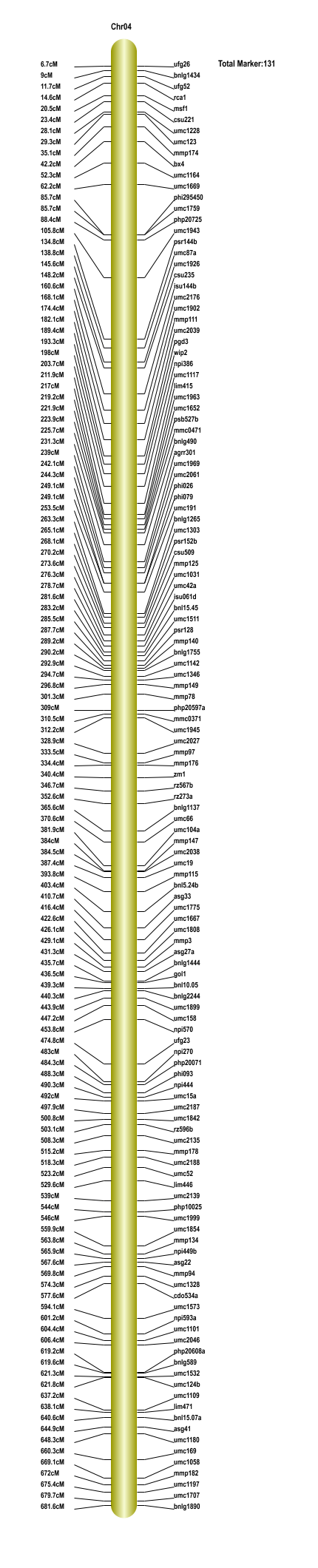

Supplement: S4 Fig — (PNG) [file pone.0152795.s004.png]

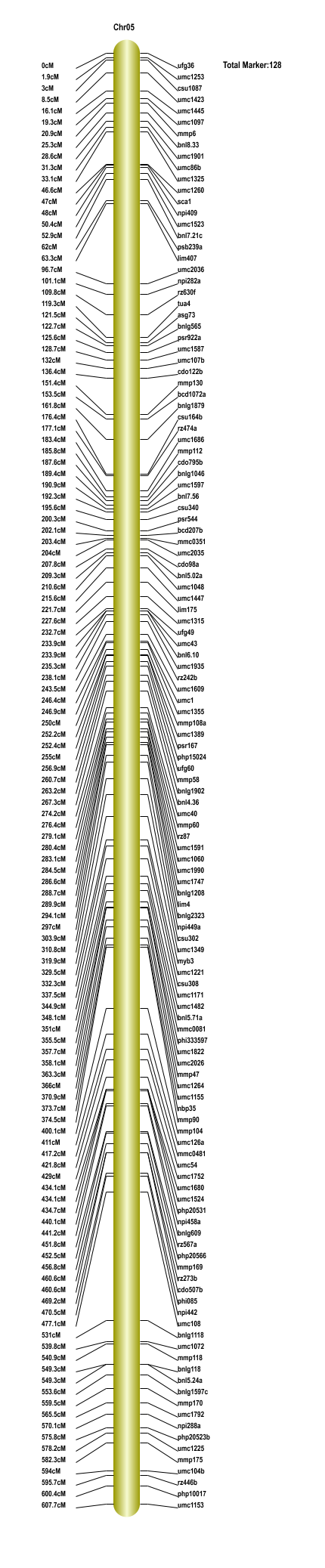

Supplement: S5 Fig — (PNG) [file pone.0152795.s005.png]

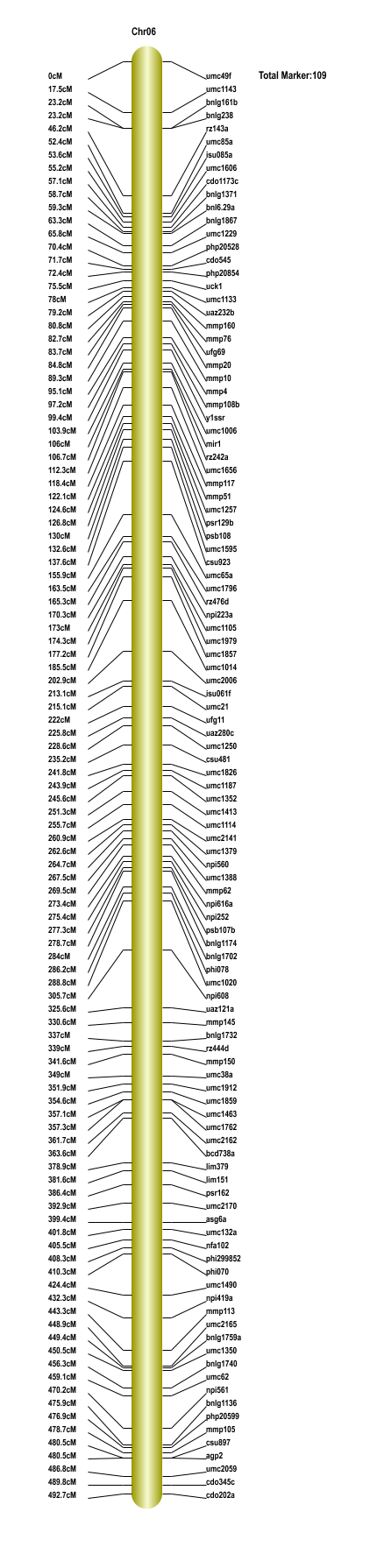

Supplement: S6 Fig — (PNG) [file pone.0152795.s006.png]

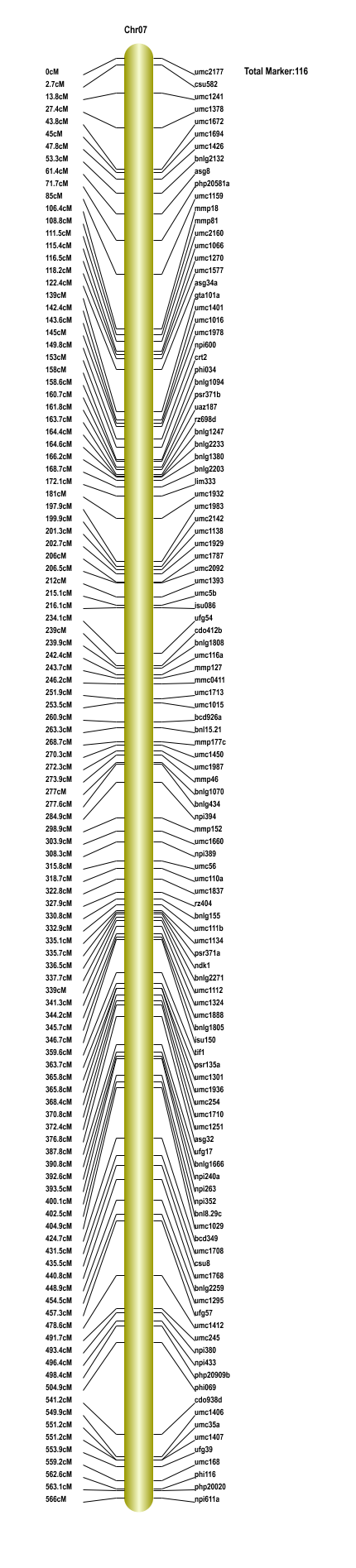

Supplement: S7 Fig — (PNG) [file pone.0152795.s007.png]

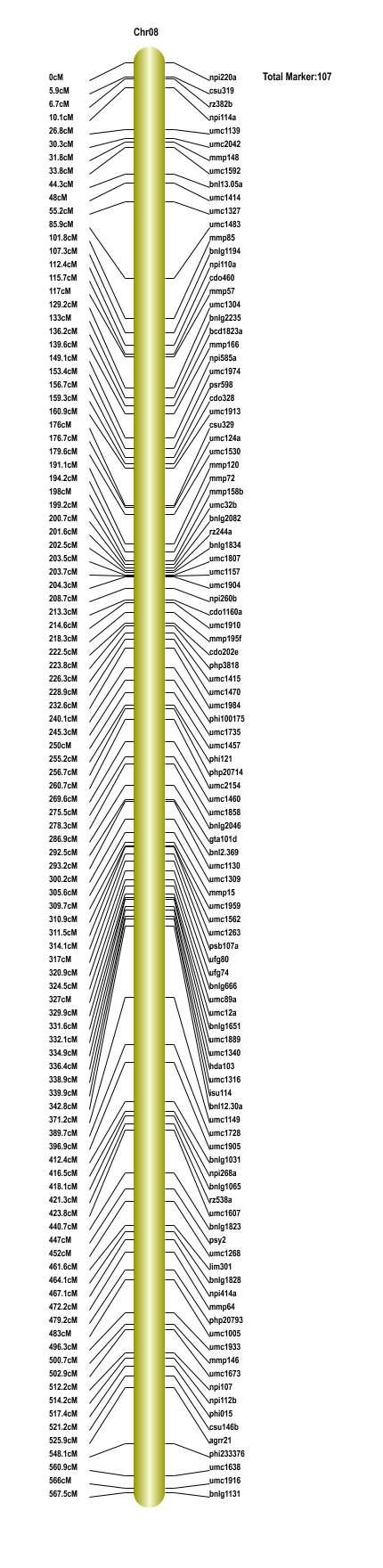

Supplement: S8 Fig — (PNG) [file pone.0152795.s008.png]

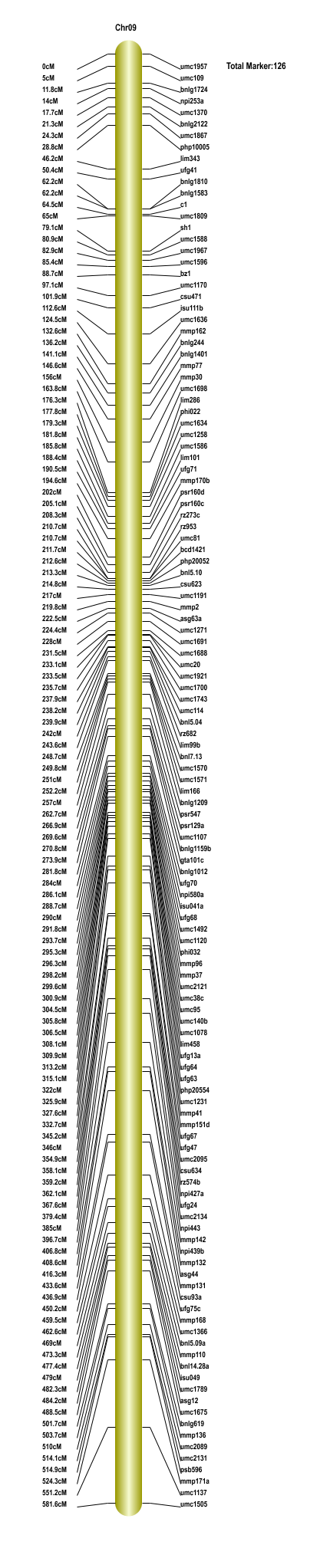

Supplement: S9 Fig — (PNG) [file pone.0152795.s009.png]

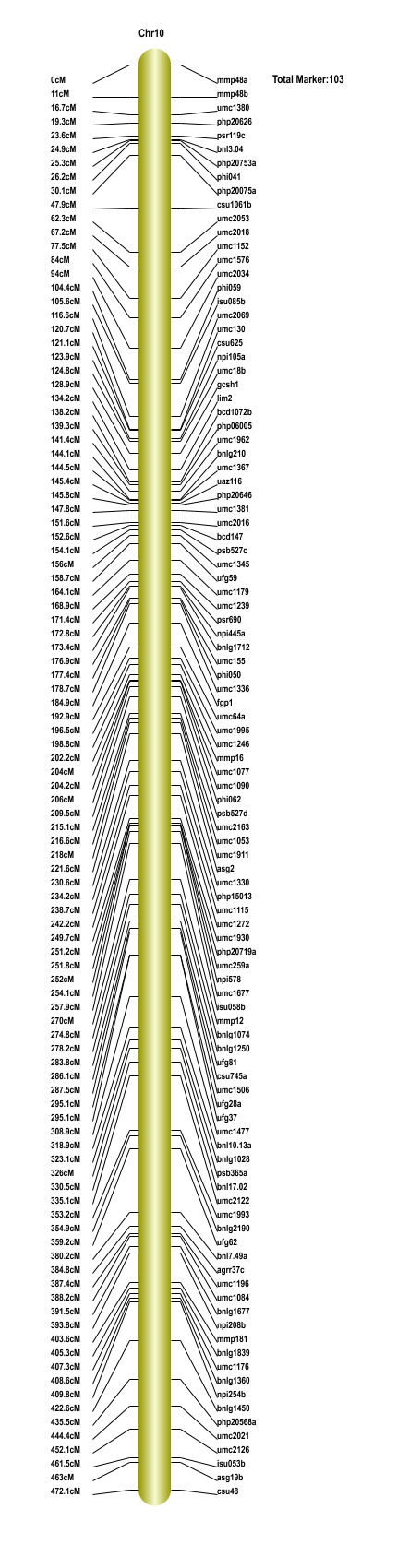

Supplement: S10 Fig — (PNG) [file pone.0152795.s010.png]
