# Supplementary material for: QTL Mapping of Low-Temperature Germination Ability in the Maize IBM Syn4 RIL Population
Source: PLoS One. 2016 Mar 31;11(3):e0152795. doi: 10.1371/journal.pone.0152795 (PMC4816396; doi:10.1371/journal.pone.0152795)
Supplement: S1 Table — (DOCX) [file pone.0152795.s011.docx]

**Table S1 The detailed procedures of QTL analysis**

| Step No. | QTL running procedures |
| --- | --- |
| Step1 | Run this command "Rmap", then choose 17, input address; choose 16, input trait name; choose 1, input "map.inp" file ; choose 5, map function; choose 1, input; choose 0, continue with these parameters. |
| Step2 | Run this command "Rcross", then choose 1, input "trait.inp" file; choose 0. |
| Step3 | Run this command "Qstats", then choose 0. |
| Step4 | Run this command "LRmapqtl", then choose 0. |
| Step5 | Run this command "SRmapqtl", then choose 0. |
| Step6 | Run this command "Zmapqtl", then choose 8-6-11-1-12-10-0 in order. |
| Step7 | Step7: Run this command "Zmapqtl", then choose 8-6-11-1-12-10-14-1000-0 in order. |
| Step8 | Run this command "Eqtl", then choose 11-PZ-0 and 9-13.8-10-1-0 in order. |
| Step9 | Run this command "Preplot", choose 12-1-0 in order. |
